# Development of a self-administered questionnaire to screen patients for cervical myelopathy

**DOI:** 10.1186/1471-2474-11-268

**Published:** 2010-11-22

**Authors:** Hiroshi Kobayashi, Shin-ichi Kikuchi, Koji Otani, Miho Sekiguchi, Yasufumi Sekiguchi, Shin-ichi Konno

**Affiliations:** 1 Department of Orthopaedic Surgery, Fukushima Medical University School of Medicine, Fukushima, Japan

## Abstract

**Background:**

In primary care, it is often difficult to diagnose cervical myelopathy. However, a delay in treatment could cause irreversible aftereffects. With a brief and effective self-administered questionnaire for cervical myelopathy, cervical myelopathy may be screened more easily and oversight may be avoided. As there is presently no screening tool for cervical myelopathy, the aim of this study was to develop a self-administered questionnaire for the screening of cervical myelopathy.

**Methods:**

A case-control study was performed with the following two groups at our university hospital from February 2006 to September 2008. Sixty-two patients (48 men, 14 women) with cervical myelopathy who underwent operative treatment were included in the myelopathy group. In the control group, 49 patients (20 men, 29 women) with symptoms that could be distinguished from those of cervical myelopathy, such as numbness, pain in the upper extremities, and manual clumsiness, were included. The underlying conditions were diagnosed as carpal tunnel syndrome, cubital tunnel syndrome, thoracic outlet syndrome, tarsal tunnel syndrome, diabetes mellitus neuropathy, cervical radiculopathy, and neuralgic amyotrophy. Twenty items for a questionnaire in this study were chosen from the Japanese Orthopaedic Association Cervical Myelopathy Evaluation Questionnaire, which is a new self-administered questionnaire, as an outcome measure for patients with cervical myelopathy. Data were analyzed by univariate analysis using the chi-square test and by multiple logistic regression analysis. According to the resulting odds ratio, β-coefficients, and p value, items were chosen and assigned a score.

**Results:**

Eight items were chosen by univariate and multiple logistic regression analyses and assigned a score. The Hosmer-Lemeshow statistic showed p = 0.805. The area under the receiver operation characteristic curve was 0.86. The developed questionnaire had a sensitivity of 93.5% and a specificity of 67.3%.

**Conclusions:**

We successfully developed a simple self-administered questionnaire to screen for cervical myelopathy.

## Background

Cervical myelopathy is caused by mechanical and dynamic compression of the spinal cord and developmental spinal canal stenosis [1]. Patients with cervical myelopathy have various symptoms, such as numbness, pain, hypoesthesia and weakness of the extremities, pain and stiffness of the neck, manual clumsiness, walking disturbance, and urinary disturbance [2-5]. Numbness of the upper extremities is one of the chief symptoms in cervical myelopathy and patients with cervical myelopathy who have this numbness frequently consult a general outpatient clinic or a primary care practitioner. Upper extremity numbness may also be caused by entrapment neuropathy, such as carpal and cubital tunnel syndromes. Therefore, diagnosis of cervical myelopathy in such patients is sometimes difficult and costly in primary care. To make an accurate diagnosis, much data must be examined, such as the case history, physical examination including a neurological examination, and imaging tests. Furthermore, misdiagnosis and delayed treatment may result in irreversible consequences, such as paralysis, urinary disturbance, and walking disturbance. Therefore, accurate diagnosis and treatment by a specialist are needed for a good outcome. If there were a brief, self-administered questionnaire for cervical myelopathy screening, patients could answer it easily while waiting for a primary care or outpatient clinic consultation. However, there is presently no scientifically established questionnaire to screen for cervical myelopathy. The aim of this study was to develop a self-administered questionnaire to screen for cervical myelopathy.

## Methods

### Ethical considerations

The present study was approved by the institutional review board of Fukushima Medical University and was conducted in compliance with ethical standards. All participants gave their written informed consent.

### Objective

One hundred eleven patients were enrolled in this case-control study (Table 1). In the myelopathy group, 62 patients (48 men, 14 women) with a diagnosis of cervical myelopathy who underwent surgery at Fukushima Medical University Hospital from February 2006 to September 2008 were included. Cervical myelopathy was secondary to a herniated disc in 5 patients, spondylosis in 32 patients, ossification of the posterior longitudinal ligament in 22 patients, and a combination of these in three patients. To develop a questionnaire to screen for cervical myelopathy, the control group comprised patients with upper extremity symptoms not caused by cervical myelopathy. Patients with dementia, a history of cervical operations, rheumatoid arthritis, infectious spondylitis, cervical myotrophy, tumor, spinal injury, cerebral infarction, and trauma were excluded.

**Table 1:** Demographic data of the Myelopathy and Control groups

	Control group (n = 49)		Myelopathy group (n = 62)	
Mean age (y (SD)	62.1 (13.3)		62.3 (13.8)	
Male (%)	20 (38.8)		48 (77.4)	
Female (%)	29 (61.2)		14 (22.6)	
Diagnosis (%)	CTS	34 (69.4)	CSM	32 (51.6)
	CuTS	9 (18.4)	CDH	5 (8.1)
	CTS + CuTS	1 (2.0)	OPLL	22 (35.5)
	CTS + DN	1 (2.0)	combination	3 (4.8)
	TTS + DN	1 (2.0)		
	TOS	1 (2.0)		
	Cervical radiculopathy	1 (2.0)		
	Neuralgic amyotrophy	1 (2.0)		

CTS: Carpal Tunnel Syndrome

CuTS: Cubital Tunnel Syndrome

DN: Diabetic Neuropathy

TTS: Tarsal Tunnel Syndrome

TOS: Thoracic Outlet Syndrome

CSM: Cervical Spondylotic Myelopathy

CDH: Cervical Disc Herniation

OPLL: Ossification of Posterior Longitudinal Ligament

### Questionnaire

There were 20 candidate items for the questionnaire (Table 2), all taken from the Japanese Orthopaedic Association Cervical Myelopathy Evaluation Questionnaire (JOACMEQ), a new self-administered questionnaire used as an outcome measure for patients with cervical myelopathy [6-10]. JOACMEQ is a questionnaire produced by identifying items regarded by specialists as important for the evaluation of cervical myelopathy and performing statistical selection. Its validity and reliability have already been demonstrated [6-9]. JOACMEQ consists of questions about the functional capacity of the cervical spine, physical function of the upper and lower extremities, and bladder function, which might be disturbed by cervical myelopathy, and items for quality of life (QOL). Because QOL items might be useless for screening, the QOL items were not included. To make the questionnaire easy for respondents to answer, the questions were changed to be answered "yes" or "no". Respondents were asked to consider their physical condition in the preceding week. If a respondent's condition had changed depending on the day or the time, the respondent was asked to identify the worst condition by circling the item number of "your worst condition". Patients in the myelopathy group answered the questionnaire a day before surgery. In the control group, outpatients satisfying the criteria answered the questionnaire prospectively in the outpatient clinic. The answers to these items were compared between the two groups.

**Table 2:** Candidates from the JOACMEQ questionnaire

Q1-1	While in the sitting position, can you look up at the ceiling by tilting your head upward?
Q1-2	Can you drink a glass of water without stopping despite the neck symptoms?
Q1-3	While in the sitting position, can you turn your head toward the person who is seated to the side but behind you and speak to that person while looking at his/her face?
Q1-4	Can you look at your feet when you go down the stairs?
Q2-1	Can you fasten the front buttons of your blouse or shirt with both hands?
Q2-2	Can you eat a meal with your dominant hand using a spoon or a fork?
Q2-3	Can you raise your arm? (Answer for the weaker side.)
Q3-1	Can you walk on a flat surface?
Q3-2	Can you stand on either leg without holding onto something? (or the need to support yourself)
Q3-3	Do you have difficulty climbing the stairs to one floor above?
Q3-4	Do you have difficulty bending forward, keeling, and stopping?
Q3-5	Do you have difficulty walking for 15 minutes?
Q4-1	Do you have urinary incontinence?
Q4-2	Do you go to the bathroom at night?
Q4-3	Do you have a feeling of residual urine in your bladder after voiding?
Q4-4	Can you initiate (start) your urine stream immediately when you want to void?
Q5	Do you have neck pain, shoulder pain, and neck stiffness?
Q6	Do you have chest tightness?
Q7	Do you have pain or numbness in the upper extremity?
Q8	Do you have pain or numbness from chest to forefoot?

### Statistical analyses

First, the chi-square test was used for univariate analysis. Odds ratios (ORs), 95% confidence intervals (95%CI), and p values were calculated. Items with ORs ≥2 and p values < 0.05 were included in the multiple logistic regression analysis. Next, β-coefficients, adjusted ORs, 95%CI, and p values were calculated. Items with ORs < 2 were removed.

Using a regression coefficient-based scoring system, a score-based questionnaire to screen patients with cervical myelopathy was developed based on the multiple logistic regression equations. The calibration was evaluated using the Hosmer-Lemeshow chi-square statistic (p > 0.05 for all models); p ≥ 0.05 supports the goodness of fit.

To generate a simple integer-based point score for each predictor variable, scores were assigned by dividing the β-coefficient by two-fifths of the sum of the two smallest coefficients in the model and rounding up to the nearest integer. The overall risk score for each patient was calculated by summing the scores of each component.

The discrimination ability of the models was assessed by the area under the receiver operating characteristic (ROC) curve. Discriminatory power is the ability to identify cervical myelopathy. An area of 1.00 under the ROC curve indicates perfect discrimination, whereas an area of 0.50 indicates complete absence of discrimination. The calibration was also evaluated by comparing the prevalence of cervical myelopathy in the score. To examine the performance of the questionnaire, the sensitivity, specificity, positive likelihood ratio, and negative likelihood ratio were calculated. This questionnaire needed a high sensitivity and a low negative likelihood ratio (< 0.1) for screening. The positivity criterion for the presence of cervical myelopathy was defined as the point with sufficiently high sensitivity for screening. All statistical analyses were performed using the SPSS software program (version 11.0.1) running on Microsoft Windows Vista (Microsoft, Redmond, WA, USA).

## Results

### Univariate analysis

The 20 items were subjected to univariate analysis and the OR was calculated for each item (Table 3). Thirteen items had an OR ≥ 2 and p < 0.05: Q1-3, Q2-1, Q3-2, Q3-3, Q3-4, Q3-5, Q4-1, Q4-3, Q4-4, Q5, Q6, Q7, and Q8. Most items representing lower leg function, bladder function, and subjective symptoms were included. In contrast, upper extremity items were not significant, which suggested that patients in both groups had upper extremity disorders. Thus, the 13 items with an OR ≥ 2 and p < 0.05 were chosen for the next step.

**Table 3:** Univariate analysis for factors from the questionnaire

	Positive rate				
		
Item	Control	Myelopathy	OR	95% CI	*p *value
Q1-1	2.08	11.29	5.98	0.71 - 50.39	0.135
Q1-2	18.75	22.58	1.26	0.50 - 3.23	0.646
Q1-3	14.58	36.07	3.30	1.27 - 8.60	0.016 *
Q1-4	8.33	16.67	2.20	0.64 - 7.51	0.255
Q2-1	14.58	32.26	2.79	1.07 - 7.30	0.044 *
Q2-2	6.25	11.29	1.91	0.47 - 7.81	0.509
Q2-3	14.58	4.84	0.30	0.073 - 1.22	0.10
Q3-1	2.08	9.68	5.04	0.59 - 43.3	0.108
Q3-2	12.50	44.83	5.69	2.09 - 19.46	< 0.001 *
Q3-3	17.02	80.65	20.31	7.57 - 54.54	< 0.001 *
Q3-4	28.26	74.19	7.30	3.10 - 17.21	< 0.001 *
Q3-5	15.22	74.19	16.02	5.98 - 42.91	< 0.001 *
Q4-1	10.64	38.71	5.31	18.40 - 15.29	0.001 *
Q4-2	61.70	73.77	1.75	0.77 - 3.96	0.213
Q4-3	23.91	55.74	4.01	1.72 - 9.33	0.001 *
Q4-4	10.64	54.84	10.20	3.56 - 29.25	< 0.001 *
Q5	51.02	89.83	8.48	3.08 - 23.36	< 0.001 *
Q6	2.08	32.76	22.90	2.93 - 178.79	< 0.001 *
Q7	66.67	98.31	29.00	3.68 - 228.87	< 0.001 *
Q8	20.83	79.66	14.88	5.80 - 38.172	< 0.001 *

*p < 0.05 and OR ≥ 2, OR: odds ratio, CI: confidence interval

### Multiple logistic regression analysis

On multiple logistic regression analysis using the 13 items (Table 4), the β-coefficients, adjusted ORs, 95%CI, and p values were calculated. Eight of the thirteen items had an adjusted OR ≥ 2: Q3-3, Q3-4, Q4-3, Q4-4, Q5, Q6, Q7, and Q8. Multiple logistic regression analysis was again performed with these eight items. For the final model, the Hosmer-Lemeshow statistic showed p = 0.805, indicating good calibration.

**Table 4:** Multivariate analysis

Item	β-coefficient	Adjusted OR	95% CI	*p *value
Q1-3	-1.79	0.17	0.009 - 3.11	0.23
Q2-1	0.482	1.619	0.13 - 19.78	0.71
Q3-2	-0.79	0.45	0.025 - 8.14	0.59
Q3-3	2.96	19.24	0.88 - 420.71	0.06 *
Q3-4	-1.04	0.36	0.15 - 8.49	0.52
Q3-5	1.78	5.93	0.52 - 67.65	0.15 *
Q4-1	0.67	1.95	0.20 - 18.59	0.56
Q4-3	1.68	5.35	0.85 - 33.57	0.07 *
Q4-4	1.83	6.26	0.63 - 62.63	0.02 *
Q5	1.68	5.35	0.62 - 46.10	0.13 *
Q6	1.82	6.11	0.39 - 96.09	0.20 *
Q7	3.24	25.62	0.019 - 35024.02	0.37 *
Q8	1.49	4.43	0.79 - 24.88	0.09 *

*OR ≥ 2

### Assignment of scores

**Figure 1:**
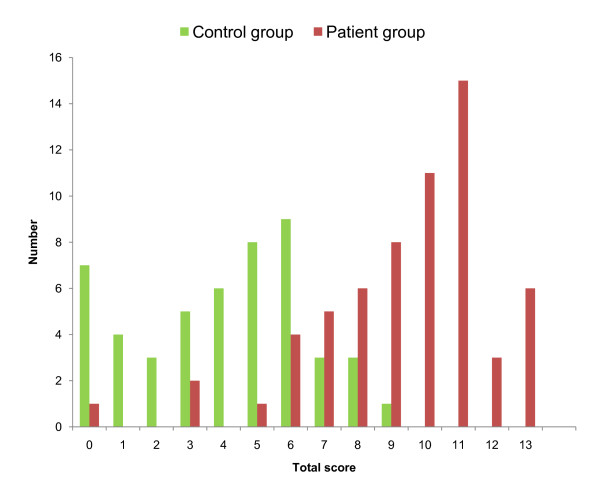
**Histogram showing the distribution of the total scores in the two groups**.

**Figure 2:**
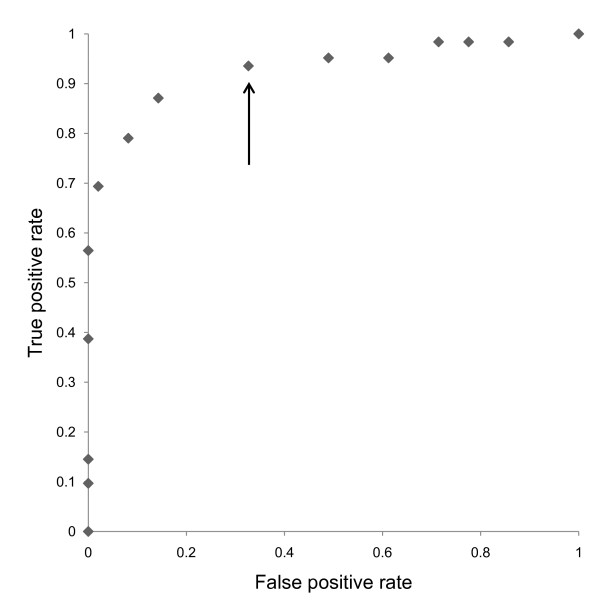
**Receiver operating characteristic (ROC) curves**. The arrow shows the cut-off point assigned to 6.

According to the calculated β-coefficients, score values were defined for the eight items. An integer score derived from the β-coefficients was assigned to the identified items (Table 5). For each patient, all applicable score values were summed up to attain a total score for the patient. The sum of the risk scores for each patient ranged from 0 to 13 (Figure 1). Finally, a self-administered questionnaire was developed (Table 6). The area under the ROC curve was 0.86; thus, the model had good discriminatory power (Figure 2).

**Table 5:** Scoring

Item	β-coefficient	Adjusted OR	95% CI	*p *value	Assigned score
Q3-3	2.06	7.88	1.20 - 51.59	0.031	2
Q3-5	1.07	2.92	0.40 - 21.16	0.29	1
Q4-3	1.41	4.09	0.89 - 18.77	0.07	1
Q4-4	1.30	3.68	0.61 - 22.28	0.16	1
Q5	1.87	6.50	0.95 - 44.31	0.056	2
Q6	1.54	4.65	0.35 - 61.71	0.24	2
Q7	2.78	16.14	0.087 - 3003.79	0.30	3
Q8	1.31	3.70	0.82 - 16.63	0.019	1

**Table 6:** Self-administered questionnaire

	Assigned score
Do you have difficulty climbing the stairs to one floor above?	2
Do you have difficulty walking for 15 minutes?	1
Do you have a feeling of residual urine in your bladder after voiding?	1
Can you initiate (start) your urine stream immediately when you want to void?	1
Do you have neck pain, shoulder pain, and neck stiffness?	2
Do you have chest tightness?	2
Do you have pain or numbness in the upper extremity?	3
Do you have pain or numbness from chest to forefoot?	1

Total score cut-off point: ≥ 6

Sensitivity: 93.5%

Specificity: 67.3%

Positive likelihood ratio: 2.96

Negative likelihood ratio: 0.096

### Setting of the cut-off point

The positivity cut-off point was defined as 6, because the sum of the sensitivities was sufficient for screening. Given that the positivity criterion for the score was greater than 6, this questionnaire had a sensitivity of 93.5%, a specificity of 67.3%, a positive likelihood ratio of 2.96, and a negative likelihood of 0.096.

## Discussion

Patients with cervical myelopathy have various symptoms, such as numbness, pain, hypoesthesia and weakness of the extremities, pain and stiffness of the neck, manual clumsiness, walking disturbance, and urinary disturbance [2-5]. Because upper extremity symptoms may be caused by other diseases including cervical myelopathy, entrapment neuropathy, and diabetes mellitus, it is difficult to distinguish between cervical myelopathy and other diseases. The clinical findings of cervical myelopathy did not have a high sensitivity, so an experienced physician was required to make the diagnosis. Cervical myelopathy is diagnosed based on the symptoms, neurological findings, and magnetic resonance images. However, there is no gold standard for cervical myelopathy. It is reported that 20% of patients with cervical myelopathy have no myelopathic signs, including Hoffmann's sign, an inverted brachioradialis reflex, clonus, and Babinski's sign [11]. Therefore, a method to easily screen for cervical myelopathy in primary care clinics is needed.

In this study, a self-administered questionnaire including eight items to screen for cervical myelopathy was developed. In the present study, the item pool was chosen from JOACMEQ, a self-administered questionnaire used as an outcome measurement for patients with cervical myelopathy, that was developed using epidemiological approaches and has been shown to have validity, reliability, and responsiveness [6-9]. JOACMEQ consists of four subgroups: function of the cervical spine, upper extremities, lower extremities, and bladder. In the questionnaire developed in this study, items for lower leg function and urination disorder were included. These facts suggests that long tract signs are important for making the diagnosis of cervical myelopathy, which is in agreement with clinical practice.

When the cut-off point for the total score was set at 6, the sensitivity was 93.5% and the specificity was 67.3%. These values are sufficiently high for screening. The positive likelihood ratio for this questionnaire was 2.96. This value means that this questionnaire is not useful for making a definitive diagnosis of cervical myelopathy. The negative likelihood was 0.096, which means that this questionnaire is useful to rule out the other diseases. Therefore, these results suggest that the questionnaire developed in this study is useful for cervical myelopathy screening.

The fact that the patient group comprised only surgical cases is one of the limitations of this study. To develop the questionnaire, only surgical cases were included in the patient group because they had a confirmed diagnosis of cervical myelopathy. Because this questionnaire is intended to be used to screen for cervical myelopathy in the primary care setting, however, a validation study using a larger group that includes mild, non-surgical cases of cervical myelopathy is required to test the questionnaire's validity and reliability. Another limitation of this study is that the control group consisted mostly of patients with peripheral entrapment neuropathy who underwent surgery; therefore, diseases of various severities should be evaluated. Despite these limitations, this new self-administered questionnaire is effective and easy to use in clinical practice. This questionnaire has only eight items that fit on 1 A4-sized page and patients can complete the questionnaire in about 5 min. We expect that the use of this questionnaire in primary care would improve the accuracy of cervical myelopathy screening. Further studies are needed to investigate the accuracy of this questionnaire in patients with cervical myelopathy of various degrees of severity.

## Conclusion

We developed a simple self-administered questionnaire to screen for cervical myelopathy in outpatients.

## Competing interests

The authors declare that they have no competing interests.

## Authors' contributions

HK, SK conceived the study and participated in the study design. HK performed the statistical analysis. HK drafted the initial manuscript for journal submission. All authors coordinated data collection. All authors read and approved the final manuscript.

## Pre-publication history

The pre-publication history for this paper can be accessed here:

http://www.biomedcentral.com/1471-2474/11/268/prepub
